# Tailoring Morphology and Vertical Yield of Self-Catalyzed GaP Nanowires on Template-Free Si Substrates

**DOI:** 10.3390/nano11081949

**Published:** 2021-07-28

**Authors:** Vladimir V. Fedorov, Yury Berdnikov, Nickolay V. Sibirev, Alexey D. Bolshakov, Sergey V. Fedina, Georgiy A. Sapunov, Liliia N. Dvoretckaia, George Cirlin, Demid A. Kirilenko, Maria Tchernycheva, Ivan S. Mukhin

**Affiliations:** 1 Nanotechnology Research and Education Centre, Alferov University, Khlopina 8/3, 194021 St. Petersburg, Russia; yury.berdnikov@itmo.ru (Y.B.); bolshakov@live.com (A.D.B.); fedina.serg@yandex.ru (S.V.F.); sapunovgeorgiy@gmail.com (G.A.S.); liliyabutler@gmail.com (L.N.D.); george.cirlin@mail.ru (G.C.); imukhin@yandex.ru (I.S.M.); 2 Institute of Physics, Nanotechnology and Telecommunications, Peter the Great Saint Petersburg Polytechnic University, Politekhnicheskaya 29, 195251 St. Petersburg, Russia; 3 Faculty of Physics, St. Petersburg State University, Universitetskaya Embankment 13B, 199034 St. Petersburg, Russia; nicksibirev@list.ru; 4 School of Physics, ITMO University, Kronverkskii, 49, 197101 St. Petersburg, Russia; 5 Ioffe Institute, Politekhnicheskaya 26, 194021 Saint Petersburg, Russia; zumsisai@gmail.com; 6 Centre of Nanosciences and Nanotechnologies, UMR 9001 CNRS, University Paris-Saclay, 10 Boulevard Thomas Gobert, 91120 Palaiseau, France; maria.tchernycheva@u-psud.fr; 7 Faculty of Chemistry, St. Petersburg State University, Universitetskaya Embankment 13B, 199034 St. Petersburg, Russia

**Keywords:** GaP, nanowires, molecular beam epitaxy, two-stage growth

## Abstract

Tailorable synthesis of III-V semiconductor heterostructures in nanowires (NWs) enables new approaches with respect to designing photonic and electronic devices at the nanoscale. We present a comprehensive study of highly controllable self-catalyzed growth of gallium phosphide (GaP) NWs on template-free silicon (111) substrates by molecular beam epitaxy. We report the approach to form the silicon oxide layer, which reproducibly provides a high yield of vertical GaP NWs and control over the NW surface density without a pre-patterned growth mask. Above that, we present the strategy for controlling both GaP NW length and diameter independently in single- or two-staged self-catalyzed growth. The proposed approach can be extended to other III-V NWs.

## 1. Introduction

A wide range of nanoscale devices from light-emitting and lasing systems to energy harvesting and sensors employ III-V nanowires (NWs) as their building blocks due to its unique morphology and outstanding electronic and optical properties [1,2]. Large surface area to volume ratio enables high sensitivity for sensor applications [1,3]. Small footprint and strain relaxation on sidewalls enable dislocation-free NW epitaxial growth on widely used Si substrates [4] and open up a route to combine materials with large lattice mismatch in axial or radial NW heterostructures [5]. In addition that, naturally smooth side facets allow NWs to serve as micro and nano cavities for both Fabry–Perot and whispering gallery modes [6,7,8], as well as low-loss optical waveguides [9].

Most device applications are sensitive to the uniformity of NW morphology and growth direction within NW arrays [2,10,11]. Generally, the ordered ensembles of vertical nanostructures with uniform shape and sizes are required for efficient light absorption and emission [12,13,14]. The latter properties are also crucial for the formation of transistor matrices [15,16], micron-pixel light emitting diodes [17] or photodetector arrays [18]. However, achieving high yield of vertical NWs on template-free nonpolar silicon wafers is challenging since it is highly dependent substrate preparation and growth conditions [19]. These factors also strongly impact the NW morphology and narrow down the window of growth conditions for NW ensembles, which are suitable for applications [19,20].

Vapor-liquid-solid (VLS) growth is the widely used approach for the synthesis of III-V NWs, which can be realized by molecular beam epitaxy (MBE) or metal organic vapor phase epitaxy (MOVPE) [10,21]. VLS growth may employ the group III (self-catalyzed approach) or foreign metal catalyst droplets. Self-catalyzed NWs are free from gold-induced deep charge trap levels which is typical for Au-catalyzed NW growth [22,23]. Moreover, it was shown that the use of metal-organic precursors can result in unintentional carbon doping and highly impact NW optical properties [24]. Thus, self-catalyzed NWs grown by solid-source MBE are promising for nanoelectronic applications, which require high purity of the III-V crystalline materials [10,25].

Gallium phosphide (GaP) is of great interest for the fabrication of nonlinear photonic components owing to the large values of the nonlinear susceptibility (χ^(2)^ ~ 70 pm/V at a wavelength around 1 µm [26]) in combination with the broad optical transparency range (0.5–11 μm [27]). Since the resonant electromagnetic properties of NWs strongly depend on their morphology, the controllable synthesis of GaP NWs is very essential for nanoscale optical devices [28]. Despite the indirect bandgap nature of GaP, recent works demonstrated quasi-direct bandgap in the GaP wurtzite phase stabilized in Au-catalyzed NWs [24,29,30] grown by the MOVPE approach allowing band structure engineering via crystal phase control [30,31]. In addition to optical properties, the work function varies significantly over the GaP crystal structure [32].

While most of the recent efforts were dedicated to finding the optimal conditions for GaAs NW growth, much less is known for the growth of GaP NWs in self-catalyzed VLS processes despite the pioneering work that was published more than five decades ago [33]. Nevertheless, several recent studies have shed light on GaP NW formation mechanism, including their growth dynamics, and have reported successful self-catalyzed growth of vertical arrays of GaP NWs with the controlled morphology on Si (111) substrates [34,35,36,37]. However, most of them report on selective area epitaxial growth requiring lithographic pre-pattering of the growth substrate. To the best of our knowledge, no previous work has reported the desired for applications simultaneous control over vertical yield and morphology of self-catalyzed GaP NWs grown on pattern-free Si substrates.

Generally, for III-V semiconductors, the lithography-free self-catalyzed NW growth on Si is extremely sensitive to the properties of the surface oxide layer. However, the specific case of GaP remains less explored in comparison with GaAs. Some works discuss the successful growth of GaAs NWs on Si substrates with native oxide layer. However, these approaches either require sophisticated substrate preparation [38,39] or may have reproducibility issues [19]. Other works rely on native oxide removal with consequent controllable regrowth of thin SiO_x_ layer [40,41,42,43]. In this work we employ wet chemical oxidation to control the properties of the obtained oxide layer on Si substrates, which then were used for MBE growth of GaP NW arrays.

We present the original approach to lithography-free growth of GaP NWs with controllable morphology. We study, in detail, the impact of Si (111) substrate preparation and MBE growth parameters in order to optimize the yield and surface density of vertical NWs. We demonstrate high diameter uniformity within NW ensembles and discuss the suppression of unwanted parasitic islands’ growth. Moreover, we describe the methods for controlling NW shape, surface density and size based on single-stage and two-stage growth processes.

## 2. Materials and Methods

### 2.1. Oxidation of Si (111) Substrates

Prior to NW growth, Si (111) wafers with 4° miscut were treated with modified RCA cleaning and Shiraki etch procedures [44,45] followed by wet chemical oxidation. Two alternative types of the oxidation procedure were employed: (i) boiling in an aqueous solution of ammonia and hydrogen peroxide NH_4_OH:H_2_O_2_:H_2_O with the volume ratio of 1:1:3 (also known as RCA-1 cleanser or “base piranha” mixture [46]) and (ii) treatment in azeotropic mixture of nitric acid HNO_3_ and water (68% of HNO_3_) at 120 °C [47,48].

### 2.2. Epitaxial Growth

The MBE growth of GaP NWs was performed via self-catalyzed (VLS) mechanism using Veeco GEN-III solid-source MBE machine (Veeco, St. Paul, MN, USA) equipped with Ga effusion cell and valved phosphorus cracker producing P_2_ molecular flux (T_cracker_ = 900 °C). The substrate temperature was controlled with a thermocouple that is calibrated by using the temperature of Si (111) 7×7↔1×1 transition as a reference [49]. We measured the beam equivalent pressure (BEP) with a conventional Bayard–Alpert vacuum gauge to monitor the group-III and group-V element fluxes. We established the stoichiometric BEP V/III flux ratio of 6 in accordance with the formation of 2D planar GaP layers on Si (001).

The oxidized Si (111) substrates were degassed in MBE load lock and buffer chambers and then annealed to create the defects in the oxide layer, which promote vertical NW growth [40,41]. The annealing carried out for 30 minutes at a temperature of 20 °C lower than one required for SiO_x_ thermal decomposition (T_dec_). In the case of nitric acid oxidation, 30 minutes of annealing at substrate temperatures higher than T_dec_ = 820 °C was required for surface oxide removal. At the same time, surface oxide prepared with the use of “base piranha” remains only until temperature reaches T_dec_ = 780 °C, which indicates a lower stoichiometry or thickness nonuniformity [44].

NW growth was initiated by simultaneous opening of Ga and P shutters. Thus, the catalytic Ga droplets were formed under P flux without Ga pre-deposition. A series of samples were synthesized at different temperature and flux ratios to investigate the mechanism of GaP NW formation.

### 2.3. Electron Microscopy Characterization

The morphology of the grown NW arrays was studied by scanning electron microscopy (SEM) using the Zeiss SUPRA 25 system (Zeiss, D-73446, Oberkochen, Germany). The NW crystal structure studies were performed by the means of transmission electron microscopy (TEM) by using the JEOL JEM–2100F field emission gun TEM (Tokyo, Japan) operating at 200 kV (with point-to-point resolution of 0.19 nm in TEM mode). Dark-field TEM images and the selected area electron micro-diffraction patterns demonstrate that NWs preserve zinc-blende structure typical for bulk GaP with a 180° rotation twinning along the [111] growth direction. The formation of thin (<200 nm) WZ-phase polytype inclusions were observed only at transient growth stages at the very beginning of NW growth and during the sample cooling under P_2_-flux. The details on TEM studies of NW crystal structure are provided in Supplementary Materials. A more detailed investigation of GaP NW crystal structure and its dependence on the growth conditions was performed using X-ray diffraction technique with an in-house X-ray source [50].

## 3. Results and Discussion

We consequently study the impact of different factors on NW growth dynamics and discuss the strategies to control the GaP NW morphology, yield of vertical NWs and their surface density on SiO_x_/Si (111) substrates. Section 3.1 discusses the crucial role of oxide layer treatment. Section 3.2 describes in detail the growth dynamics and temporal evolution of the NW shape. The impact of V/III flux ratio and substrate temperature on NW morphology and yield of vertical NWs are studied in Section 3.3 and Section 3.4, respectively, Finally, Section 3.5 shows how the limitations of a single-stage growth process can be overcome within a two-stage approach with variation of the MBE growth parameters during the process. Furthermore, we consider the above-mentioned factors in order of their importance for tailoring the properties of the obtained NWs.

### 3.1. Role of Silicon Oxide Preparation

The yield of vertical GaP NWs and the undesired formation of GaP nanoislands are found to be highly sensitive to the substrate preparation procedure. Indeed, the structural properties of the oxide layer are widely known to be highly sensitive to the annealing regime [47,48]. Previous studies of GaAs NW growth on oxidized Si substrates have shown that the formation of pinholes in the oxide layer is generally required for vertical NW growth [40,41]. This process was reported to be dependent on surface roughness, oxide thickness and contact angle of Ga droplet, which are all determined by the oxidation regime.

Here we compare substrate treatments in the “base piranha” mixture and in nitric acid in their impact on the NW growth. For each of the methods, we study a series of samples annealed at different temperatures prior to the NWs growth. It has been observed experimentally that the optimal annealing temperature T_an_ providing the highest NW yield is close to the temperature of oxide decomposition T_dec_.

**Figure 1:**
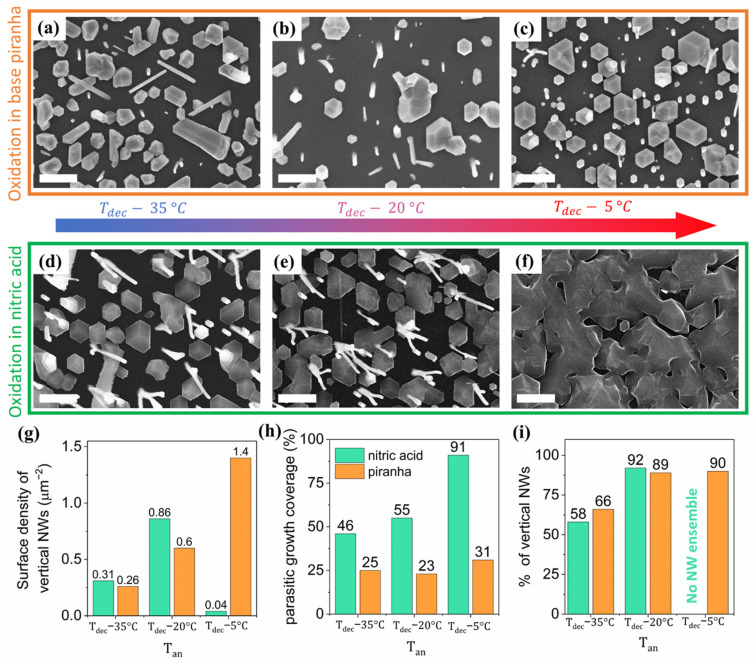
GaP NW arrays grown on an annealed SiO_x_/Si (111) surface with temperature gradient after treatment in “base piranha” (**a**–**c**) and nitric acid (**d**–**f**). The scale bars are 2 µm. T_dec_ was estimated to be 780 °C for “base piranha” and to 820 °C for nitric acid. Comparative statistical analysis provides the surface density of vertical NWs (**g**), fraction of substrate area covered by parasitic islands (**h**) and fraction of vertical NWs (**i**) for annealing at temperatures 5, 20 and 35 °C below T_dec_.

In order to illustrate the effect of annealing temperature, the samples with artificially elevated temperature gradient are studied. Switching off one section of the two-zone heater resulted in the temperature variation in the range of 5 to 35 °C below T_dec_ across the wafer radius. The SEM images in Figure 1a–f illustrate the influence of the annealing temperature variation within a 3 inch substrate. Generally, the NW growth on substrates oxidized in “base piranha” is found to be less sensitive to the temperature gradient (Figure 1a–c) in comparison with the growth on wafers processed in nitric acid (Figure 1d–f). Figure 1d shows that the annealing at a temperature 35 °C below T_dec_ results in inclined growth of the large fraction of NWs. On the other hand, the growth of parasitic islands and the suppression of the NW formation are observed after annealing at temperatures about 5 °C lower than T_dec_ (as shown in Figure 1f). It should be noted that further increases in the annealing temperature for the “base piranha” oxidized substrates results in rapid oxide decomposition followed by the formation of a GaP layer similar to the one observed in Figure 1f.

Figure 1g summarizes variation of the surface density of vertical NWs with annealing temperature for both oxidation techniques. Generally, the NW surface density increases with the increase in annealing temperature until the oxide layer is heavily degraded. Simultaneously, we observe the intensification of the parasitic growth, which is illustrated in Figure 1h. We associate these effects to more intensive oxide desorption or defect formation in the oxide layer at higher annealing temperatures. In the case of nitric acid oxidation followed by annealing 5 °C below the optimal temperature, the parasitic structures cover more than 90% of the substrate area which, of course, suppresses the growth of NWs (as shown in Figure 1f). By contrast, the parasitic growth remains much less pronounced when the “base piranha” is used for the oxidation of Si (111) substrate.

The inclined growth of GaP NWs is rare after annealing at temperatures 20 °C below or closer to T_dec_. In this case, above the 90% of the NWs, they are are observed to grow vertically, as summarized in Figure 1i. However, the annealing at lower temperature reduces the fraction of vertical NWs to about 60% for both oxidation techniques. These results are original for GaP NWs growth on lithography-free although they are qualitatively consistent with the earlier observations on the growth of self-catalyzed GaAs NWs on SiO_x_/Si (111) substrates [40,41].

Thus, both approaches to oxidation of Si (111) surface provide high surface density and a large fraction of vertical NWs with limited formation of parasitic islands when T_an_ is 20 °C lower than T_dec_. The corresponding temperatures of 760 °C for “base piranha” and to 800 °C for nitric acid are considered as optimal annealing temperature and used in further growth experiments. In the case of “base piranha” oxidation, even though the maximal NW surface density was observed after annealing at T_an_ just 5 °C below T_dec_, this temperature cannot be considered as optimal due to its proximity to T_dec_ within the uncertainty in measurement of substrate temperature. We also note that minimal parasitic growth is observed after “base piranha” oxidation and annealing at 760 °C (T_dec_—20 °C ). As we show in the following discussion, the parasitic growth can be further suppressed by varying the conditions of GaP NW growth.

### 3.2. Roles of the Growth Temperature and Flux Rates

**Figure 2:**
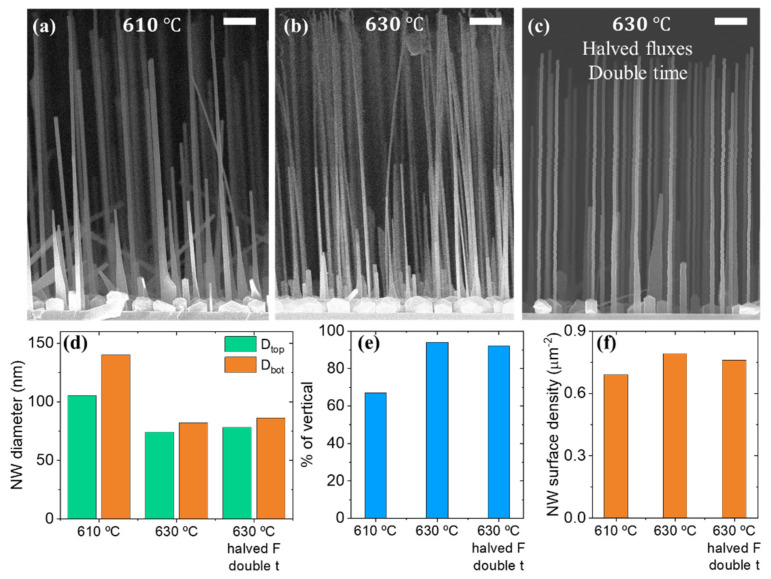
Side-view SEM images of GaP NW arrays grown at T_gr_ = 610 °C (**a**), T_gr_ = 630 °C (**b**) and at halved Ga molecular fluxes with the V/III flux ratio of 24 (**c**). The scale bars are 1 µm. The diagrams compare mean values of NW top (D_top_) and bottom (D_bot_) diameters (**d**), fraction of vertical NWs (**e**) and NW surface density (**f**) for the growth at elevated temperature and halved group V and III fluxes.

In this section, we discuss the impact of growth temperature T_gr_ lying in a narrow range centered at 610 °C on the NW morphology and yield of vertical NWs. Figure 2 show SEM images together with results of statistical analysis illustrating the effect of the growth temperature on the formation of GaP NWs on substrates treated in nitric acid. Elevation of the growth temperature by 20 °C from 610 °C results in the decrease in NW top diameter, as shown in Figure 2d. The NW length is found to vary insignificantly whether Ga and P molecular fluxes are kept constant. Simultaneously, we observe less pronounced tapering at the elevated growth temperature.

Whilst the NW surface density remains at the level of 0.6–0.9 µm^−2^, the fraction of vertically oriented NWs significantly increases from 70% to 90%, with the increase in the growth temperature from 610 to 630 °C, which is illustrated in Figure 2e. We assume that the high growth temperature promotes the formation of non-wetting catalytic Ga droplets with a proper contact angle, which results in the growth of vertical NWs [51].

In order to distinguish the effects of growth temperature and flux rates on NWs’ formation, we synthesized a sample with halved Ga and P flux rates and doubled growth duration while keeping an identical substrate temperature of 630 °C and surface oxide treatment and V/III ratio equal to 24. Figure 2c demonstrates a typical SEM image of the obtained NWs. The growth at halved fluxes and doubled duration results in the slight shortening of the mean NW length, which corresponds to an almost two-fold reduction in the NW growth rate from 160 to 80 nm/min for halved fluxes. In the meantime we observed almost no difference in diameters at the top of NWs, as shown in Figure 2d. Thus, the NW growth rate can be controlled independently from NW diameter via changing both growth fluxes at a fixed V/III ratio.

It is worth noting that the reduced formation of parasitic nanoislands is observed after halving the growth fluxes. Qualitatively, this effect is visible in SEM images presented in Figure 2b,c. From SEM images in Figure 2a,b we also note that the NWs grown at T_gr_ = 610 °C are slightly tapered, while the structures grown at T_gr_ = 630 °C possess almost vertical sidewalls. In the next section we study the NW shape in detail by analyzing the NW morphology evolution during the growth.

### 3.3. NW Growth Evolution

We study the NW morphology evolution and NW surface density within a series of samples with similar substrate preparation and the same growth conditions (T_gr_ = 630 °C and V/III ratio of 24), but for different growth times *t* varying from 60 to 300 min. We characterize the NW morphology evolution by temporal dependences of NW length L and diameters at the top $D_{top}$ and bottom $D_{bot}$. The data in Table 1 summarizes the mean values and standard deviations of L, $D_{top}$ and $D_{bot}$ over the NW ensembles.

**Figure 3:**
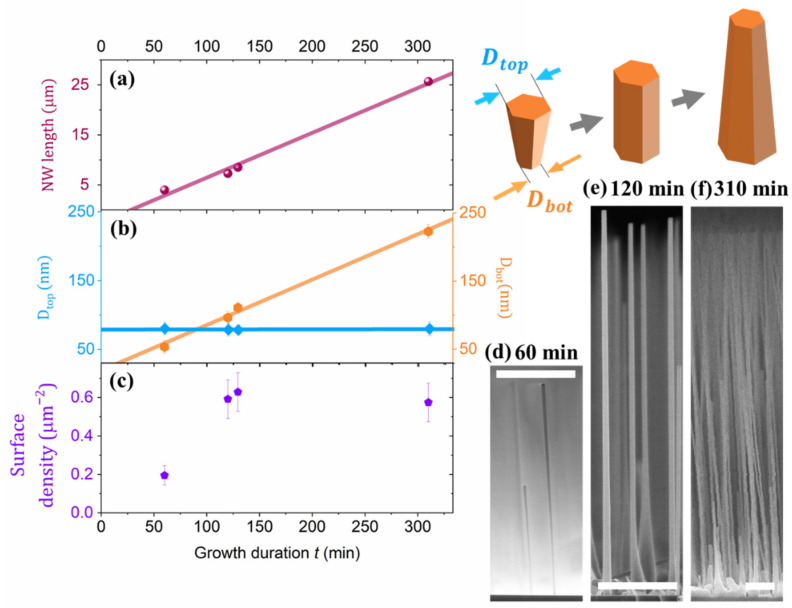
Temporal evolution of the NW mean length (**a**), top and bottom diameters (**b**) and surface density (**c**). SEM images show the side-view images of GaP NWs grown for 60 (**d**), 120 (**e**) and 310 min (**f**). The scale bars are 2 µm.

During the growth, the NW length L increases at a constant rate around 80 nm/min, as shown in Figure 3a. In the meantime, we observed the change of the NWs’ shape from inverse tapering after 60 min of growth to tapering in longer epitaxial processes (see Figure 3b).

The inverse tapering of the NWs grown for *t* = 60 min or less (see Figure 3d) can be attributed to the expansion of Ga droplet at the early stage of the growth. However, in longer growths, we observe the stabilization of $D_{top}$ around 80 nm at a V/III ratio of 24 (as shown in Figure 3b and in the second column of Table 1). Meanwhile $D_{bot}$ linearly increases with *t* and, thus, the NW morphology subsequently evolves from inverse tapered to straight and finally to tapered shape.

Figure 3c shows the variation of the NW surface density with an increase in the growth duration. An important observation is the increase in the NW surface density between *t* = 60 and 120 min. Therefore, the nucleation of GaP NWs continues even after an hour of growth under the considered MBE conditions.

Next, we introduce the growth model to interpret the observed effects. In Ga-catalyzed VLS growth, NW top diameter is proportional to the size of the droplet [52], which almost entirely consists of gallium. The evolution of the droplet volume V is governed by the rate of direct impingement of Ga adatoms and their diffusion flux from NW sidewalls to the droplet and crystallization rate dL/dt can be described as follows:

(1) $$\frac{dV}{dt}=\pi D_{top}^{2}F_{Ga}f\left(\theta ,\varphi \right)/4+F_{Ga}D_{top}\lambda \;tan\left(\varphi \right)-\frac{dL}{dt}\pi D_{top}^{2}/4$$

where $F_{Ga}$ is the Ga flux in terms of the deposition rate measured in nm/s and λ is the mean free path of Ga adatoms on the NW sidewalls. *f(θ,φ)* is the ratio of the droplet and NW top facet cross-section areas, which is a function of the droplet contact angle *θ* and a beam incidence angle *φ* [52]. At a stable contact angle *θ*, the droplet volume can be calculated as $V=g\left(\theta ,\varphi \right)D_{top}^{3}/$12, where $g\left(\theta ,\varphi \right)$ is the geometric factor [52]. Then, according to Equation (1), the evolution of NW top diameter is described as follows.

(2) $$g\left(\theta \right)\;\frac{dD_{top}}{dt}=F_{Ga}f\left(\theta ,\varphi \right)+\frac{4}{\pi }F_{Ga}\frac{\lambda }{D_{top}}tan\left(\varphi \right)-\frac{dL}{dt}$$

In self-catalyzed process, the axial growth rate is limited by group V flux to the droplet $F_{P}$ and, thus, $dL/dt\approx k\;f\left(\theta ,\varphi \right)F_{p}$. Here, the coefficient k accounts for the direct and re-adsorption transport of group V atoms into the droplet [36,53]. Within the considered series of growth processes, we kept constant $F_{P}$ with various durations.

Previous studies of GaAs NW growth have shown the stabilization of the droplet sizes in NW ensembles growing with purely gallium and Ga-rich AuGa droplets^28–30^. However, this effect, which is also observed in our experiments, was not widely discussed in the case of GaP NWs. The volume and top diameter stabilization condition $D_{top}=D_{top}^{*}=const$ requires $dD_{top}/dt=0$ and, thus, according to Equation (2) yields the following:

(3) $$D_{top}^{*}=\frac{4\lambda \;tan\;\left(\varphi \right)\;}{\pi f\left(\theta ,\varphi \right)\left(kf_{V/III}-1\right)},$$

where $f_{V/III}$=$F_{P}$/$F_{Ga}$ and the following is described.

(4) $$\frac{dL}{dt}=F_{Ga}f\left(\theta ,\varphi \right)+\frac{4}{\pi }F_{Ga}\frac{\lambda }{D_{top}^{*}}tan\left(\varphi \right)=const.,$$

Assuming that the growth duration Δt is much longer than the duration of the nucleation stage, we neglect variation of the growth rate at the initial stage and, thus, the NW length L increases linearly with the growth duration.

(5) $$L\approx \left(F_{Ga}f\left(\theta ,\varphi \right)+\frac{4}{\pi }F_{Ga}\frac{\lambda }{D_{top}^{*}}tan\left(\varphi \right)\right)\Delta t.$$

Considering $F_{Ga}$ and $f\left(\theta ,\varphi \right)$ at experimentally defined θ=120° and φ=34°, we use the slope of $L\left(t\right)$ temporal dependence in Equation (5) to estimate λ as follows.

(6) $$\lambda =D_{top}^{*}\frac{\pi }{4tan\left(\varphi \;\right)}\left(\frac{L/\Delta t}{\;F_{Ga}}-f\left(\theta ,\varphi \right)\right)$$

One can note that Equation (6) provides the estimation for λ deduced from the experimentally observed values. Our analysis provides lower estimated values of λ for higher substrate temperatures, which we relate with more intense desorption of Ga adatoms from the NW sidewalls at higher temperatures^14^. The shortening of λ results in lower diffusion flux of Ga adatoms and, therefore, reduces the droplet volume together with stable NW top diameter in accordance with Equations (1)–(4).

Thus, the stabilization of adatom fluxes results in the stabilization of the droplet volume and constant axial growth rate for NWs longer than the diffusion length for Ga adatoms on the NW sidewalls. The NW shape evolves from inverse tapering to tapering due to the continuation of the radial growth after saturation of the droplet volume (thus, the stabilization of $D_{top}$).

According to Equation (2), one can assume that the stable value $D_{top}^{*}$ is dependent on growth temperature and V/III ratio. In the next subsection, we discuss the role of V/III flux ratio in detail.

### 3.4. Role of the V/III Ratio

In this subsection we discuss the influence of group V/III molecular beam flux ratio on the growth of GaP NWs. We extend the theoretical approach of the previous section to the case of variable V/III ratio and verify the results with the experimental observations within a series of samples grown at T_gr_ = 610 °C, where we varied V/III ratio from 12 up to 30 by tuning the phosphorus flux, while keeping Ga-flux constant and corresponding to the nominal planar GaP growth rate of 6.33 nm/min.

**Figure 4:**
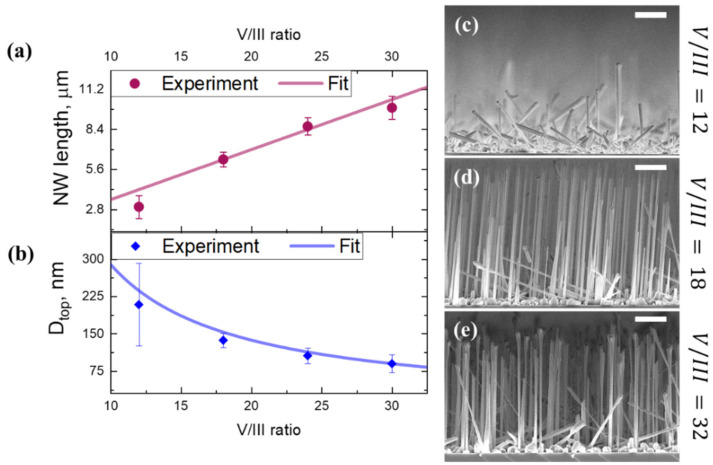
Dependences of the mean NW length (**a**) and top diameter (**b**) on the V/III flux ratio (Ga flux is kept constant) and (**c**–**e**) side-view SEM images of a GaP NW on Si (111) grown at different V/III ratios. The scale bars are 2 μm.

Figure 4 presents the analysis of NW morphology: panels (a) and (b) provide the NW length and diameter dependences on V/III flux ratio, along with the side-view SEM images of the NW ensembles grown at $f_{V/III}=12$, 18 and 30 in panels (c), (d) and (e), respectively.

NW length increases while the NW top diameter decreases when the V/III ratio rises from 12 to 30. These experimentally obtained dependences are consistent with the theoretical curves provided by Equations (3)–(5), as shown in Figure 4a,b. In Figure 4c, one can note a rather misoriented NW growth at the lowest of V/III ratio values of 12. This sample shows the largest observed dispersion of NW diameters and lengths (ΔL/L~20%). The inverse tapering of NWs grown at $f_{V/III}=12$ witnesses the unsaturated inflation of catalytic droplet after 60 min of growth for both vertical and inclined NWs. Much more uniform NW arrays are formed at V/III of 18 and higher (ΔL/L~10%), which may indicate both homogeneous nucleation and droplets stabilization in the NW ensemble.

The SEM image in Figure 4e illustrates the case of further increases in V/III ratio beyond 30, which results in droplet consumption and interruption of the VLS growth. The decrease in NW length and an increase in their diameter due to vapor-solid radial growth are observed together with loss of the NW size homogeneity.

Therefore, the V/III flux ratio is the limiting factor for the control over diameters in ensembles of the self-catalyzed GaP NWs. For too low $f_{V/III}$ values, the droplet stabilization is slow and may be longer than the growth duration. For too high $f_{V/III}$, the VLS growth is limited by the droplet consumption. Nevertheless, we achieve control over NW stable diameter at V/III flux ratios between 18 and 30.

### 3.5. Two-Staged Growth

As shown in the previous sections, the growth at a substrate temperature of 630 °C and V/III flux ratios above 18 results in desirable yield maximization for vertical NWs together with the reduction in the stable NW diameter. The increase in diameter can be achieved by lowering V/III flux ratios, which also decreases the surface density and fraction of vertical NWs. However, a number of emerging nanophotonic applications require high yield of vertical NWs and a controllable large subwavelength-diameter [54] simultaneously, which is incompatible with the abovementioned growth process. In this section we show how to overcome this inconsistency within a two-stage growth process.

Within a two-stage growth, NWs nucleate and grow at high $f_{V/III}$ in order to obtain high yield of vertical NWs during the first stage. At the second stage, the growth is carried on at a low V/III ratio, which as we have shown previously, increases the NW stable top diameter.

**Figure 5:**
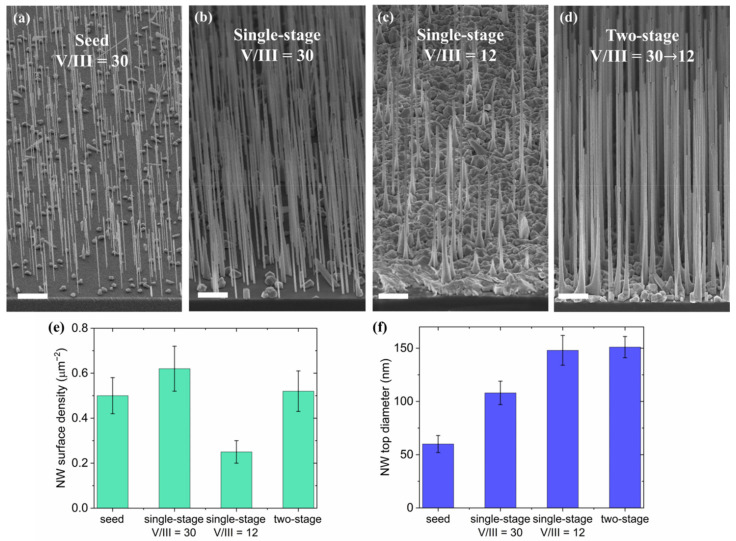
A 30° tilted view SEM images of the array of NW seeds (**a**), two-staged sample (**b**) and two reference single stage samples grown at V/III ratio of 30 (**c**) and 12 (**d**); the scale bars are 2 μm; diagrams for NW surface density (**e**) and NW top diameter (**f**) in the four samples.

In our approach the first (“Seed”) stage of the NWs growth lasts for 33 min at the V/III flux ratio of 30 and the substrate temperature of 630 °C. The SEM image in Figure 5a shows the GaP NWs obtained after the growth interruption at the end of the first stage. The transition to the second stage of the growth with low V/III ratio implies a smooth 2.5-fold increase in Ga flux from 3 nm/min up to 7.5 nm/min within 200 s, while keeping the phosphorus flux and growth temperature constant without interruption of the growth. We keep T_gr_ of 630 °C at the second stage to avoid intensive nucleation of parasitic 2D after the increase in Ga flux. Figure 5d shows a typical SEM image of GaP NW grown in the two-staged process with the second stage lasting 83 min. We note that parasitically grown islands still have not coalesced and do not form a continuous layer.

We compare the two-stage growth results with a pair of single-stage reference samples grown for 117 min under conditions identical to the first (high V/III flux ratio of 30) and second (low V/III flux ratio of 12) growth stages, illustrated in Figure 5b,c, respectively. In comparison with single stage grown at V/III = 30, the two-stage grown NWs show the mean top diameter increase from 100 to 150 nm, while widening at the NW bottom part has appeared and the parasitic growth has been more pronounced. Both the single-stage growth at high V/III ratio of 30 and the two-staged maintain a surface density very similar to the “Seed” sample (see Figure 5e), while the two-stage approach permits the increase in NW diameter by more than 40%.

NW ensembles grown in two-staged process and at low V/III ratio have similar NW top diameters of about 150 nm. This observation experimentally confirms the described theoretically stabilization of the droplet size governed by V/III flux ratio. Meanwhile, in comparison to single-stage growth at V/III = 12, the two-stage sample shows reduced parasitic growth, higher surface density and vertical sidewalls with widening at the bottom instead of pronounced tapering running along the whole length.

Thus, the two-stage growth allows self-catalyzed GaP NWs to inherit the high surface density and low parasitic growth of the first stage and to attain wide diameters without sidewall tapering at the second stage. In combination with the optimal substrate, oxidation one obtains GaP NW ensembles with high vertical yield and suppressed parasitic growth simultaneously with controllably large NW diameters compatible with optical applications.

## 4. Conclusions

We have presented an extensive study of the GaP NWs self-catalyzed MBE-grown on template-free Si (111) substrates. We demonstrate that wet chemical oxidation of Si substrate followed by the pre-growth high-temperature annealing has a decisive impact on the surface density and yield of vertical GaP NWs. Simultaneously, the morphological parameters (namely, NW top diameter and tapering) are more sensitive to the growth duration, V/III ratio and growth temperature. The latter, however, also affects the surface density of vertical NWs and could be tuned to reduce the surface density of parasitic islands. The presented lithography-free procedure of substrate preparation based on the use of “base piranha” provides the yield of vertical NWs above 90% with surface densities about 1 NW per µm^2^.

We achieved the highly uniform GaP NW ensembles via the size of Ga droplet (and, thus, the NW top diameter) saturation at the stable value. According to the developed theoretical model and the acquired experimental data, the stable top NW diameter was found to be dependent mainly on the V/III ratio, rather than on the absolute values of growth fluxes. Within this approach, we have demonstrated that the NW diameter and length can be controlled independently by choosing proper P/Ga flux ratio and growth rate, respectively.

Moreover, it was experimentally shown that the elevation of growth temperature by 20 °C results in stable GaP NW diameter decrease by about 20% and suppresses the parasitic GaP layer growth and NW radial overgrowth. This growth process provides highly homogeneous in diameter ensembles of thin NWs with almost no tapering when the droplet size is stable.

We have found that the increase in vertical yield and control of the NW diameter cannot be achieved simultaneously within a single-stage growth. Furthermore, we have demonstrated that this restriction can be overcame in a two-stage approach with the decrease in V/III flux ratio at the second growth step following the formation of NW stems at higher V/III ratio and growth temperature. In this case, the subsequent expansion of the catalyst droplet results in the increase in NW lateral size, while the radial growth on the NW side facets allows obtaining NWs with almost vertical sidewalls. We believe that the proposed approach paves the path for the growth of NWs with controllable morphology, which is very essential for various applications.

## Figures and Tables

**Table 1:** Parameters of the NW ensembles.

t (min)	$D_{top}\;(\mathrm{nm})$	$D_{bot}\;(\mathrm{nm})$	L (µm)	Estimated λ (µm)
60	80 ± 10	54 ± 9	4.00 ± 0.36	2.15
120	79 ± 10	97 ± 15	7.33 ± 0.54	2.11
130	78 ± 10	111 ± 14	8.54 ± 0.6	2.09
310	82 ± 21	216 ± 45	25.69 ± 0.6	2.19

## Data Availability

The data presented in this study are available upon request from the corresponding author.

## Supplementary Materials

The following are available online at https://www.mdpi.com/article/10.3390/nano11081949/s1, detailed information on transmission electron microscopy analysis of nanowire crystal, Figure S1: TEM of the GaP NWs grown at the different growth conditions and transferred to a to carbon-coated copper TEM grid (a), (b) Dark-field TEM images with a WZ-diffraction contrast and (c) bright-field TEM images of the GaP NW grown via the proposed two-staged approach; (d–i) selective area electron diffraction patterns obtained from the NW areas with twinned ZB (f–i), WZ (d) and mixed crystal phase (e) structure schematically depicted with dashed lines in (a–c). Figure S2: HRTEM images of the GaP NW demonstrating the atomic structure of planar defects. Twinning boundaries (TBs) and stacking faults (SFs) are marked by the white and red arrows, correspondingly.
