# Supplementary material for: Tailoring Morphology and Vertical Yield of Self-Catalyzed GaP Nanowires on Template-Free Si Substrates
Source: Nanomaterials (Basel). 2021 Jul 28;11(8):1949. doi: 10.3390/nano11081949 (PMC8400893; doi:10.3390/nano11081949)
Supplement: Supplementary file 1 [file nanomaterials-11-01949-s001.zip › nanomaterials-1292197-supplementary.pdf]

Supplementary Materials

Tailoring Morphology and Vertical Yield of Self-Catalyzed GaP Nanowires on Template-Free Si Substrates

Vladimir V. Fedorov [1,2,*], Yury Berdnikov [1,3], Nickolay V. Sibirev [3], Alexey D. Bolshakov [1,4], Sergey V. Fedina [1], Georgiy A. Sapunov [1], Liliia N. Dvoretckaia [1], George Cirlin [1], Demid A. Kirilenko [5], Maria Tchernycheva [6] and Ivan S. Mukhin [1,3,7]

[1] Nanotechnology Research and Education Centre, Alferov University, Khlopina 8/3, 194021 St. Petersburg, Russia; yury.berdnikov@itmo.ru (Y.B.); bolshakov@live.com (A.D.B.); fedina.serg@yandex.ru (S.V.F.); sapunovgeorgiy@gmail.com (G.A.S.); liliyabutler@gmail.com (L.N.D.); george.cirlin@mail.ru (G.C.); imukhin@yandex.ru (I.S.M.)
[2] Institute of Physics, Nanotechnology and Telecommunications, Peter the Great Saint Petersburg Polytechnic University, Politekhnicheskaya 29, 195251 St. Petersburg, Russia; burunduk.uk@gmail.com (V.V.F.)
[3] Faculty of Physics, St. Petersburg State University, Universitetskaya Embankment 13B, 199034 St.Petersburg, Russia; nicksibirev@list.ru (N.V.S.)
[4] School of Physics, ITMO University, Kronverkskii, 49, 197101, St. Petersburg, Russia
[5] Ioffe Institute, Politekhnicheskaya 26, 194021 Saint Petersburg, Russia; zumsisai@gmail.com (D.A.K.)
[6] Centre of Nanosciences and Nanotechnologies, UMR 9001 CNRS, University Paris-Saclay, 10 Boulevard Thomas Gobert, 91120 Palaiseau, France; maria.tchernycheva@u-psud.fr (M.T.)
[7] Faculty of Chemistry, St. Petersburg State University, Universitetskaya Embankment 13B, 199034 St. Petersburg, Russia;

* Correspondence: burunduk.uk@gmail.com

NW Orientation and Crystal Structure

An in situ analysis of the NW crystal structure by reflection high energy electron diffraction (RHEED) demonstrates that NWs grow epitaxially and preserves GaP bulk zinc-blende structure and keeps the same crystallographic orientation as the Si (111) wafer with a 180° rotation twinning along the [111] growth direction. For the vicinal substrates, the direction of NW growth is not determined by the surface normal, but aligned along the [111] Si. Thus, NW grown on the vicinal Si (111) wafers with a 4° miscut appears in SEM images to be tilted by 2° or 4° depending on the sample cleavage for SEM studies. It was also noted that under the specific growth conditions followed by the catalytic droplet consumption, the mixed zinc-blende (ZB)/wurtzite (WZ) character of the RHEED can be observed [1].

Detailed analysis of the NW structure was performed by using high-resolution transmission electron microscopy (HR-TEM) of the GaP NW formed at various growth conditions. For TEM studies, the NWs were "dry-transferred" to a carbon coated TEM grid by gently rubbing the grown sample against the TEM grid. In Figure S1a,b, one can find typical dark-field TEM (DF-TEM) images with a diffraction contrast obtained by choosing WZ Bragg reflection position. In these conditions, the bright contrast in DF-TEM images may correspond to the presence of prolonged WZ-phase segments or planar defects, namely stacking faults (SFs), and twin boundaries (TBs), which is equivalent to three or two bilayers of WZ-phase stacking sequences, respectively. From the obtained DF-TEM images and the selected area electron micro-diffraction (SAED), it can be judged that NWs possess mostly ZB structure with a high density of SFs and TBs appearing as thin bright lines crossing the NW. Twinned [110] zone axis patterns for the ZB-phase with a 180° rotational twinning along the <111> growth direction can be found in the SAED patterns presented in the Figure S1e–f, h–i. Elongation of the Bragg peaks in normal to the defect plane direction can be observed in the SAED pattern of highly defective NW region presented in the Figure S1 g, which is common for a high density of planar defects. HRTEM image with the atomic resolution presented in Figure S2 demonstrates that the discussed planar defects are mainly consists of {111} TBs with a few numbers of SFs. Thin (up to 200 nm) axial inclusions (bright contrast) with a WZ structure can be noted at the NW bottom and top parts and corresponding SAED patterns are presented in the Figure S1 d and e. Reflex positions for the ZB and wurtzite phases are marked with dashed blue (ZB) and red (ZB-twin) and solid white circles (WZ). We assume that the formation of the WZ phase in the top part of the NW is associated with the consumption of a catalytic Ga droplet during the sample cooling under the excess phosphorus flux and subsequent decrease in the contact angle favoring the nucleation at the triple line [1]. We also assume that the wurtzite inclusions in the NW bases can be formed during transient stage of catalytic droplet expansion within the specific range of wetting angles.

It is worth noting that we are not able to find the growth conditions necessary to suppress the formation of rotational twins and the thickness of defect free segments only slightly depends on the V/III flux ratio or growth

temperature. However, we found that the pronounced dependence of the planar defect density on the distance from the NW/Si (111) interface, which increases as the NW grows and becomes higher to its top. Thus, depending on the position, defect free segment can vary in its thickness from 110 nm in the middle of the NW length to 100–500 nm at the base. The effect is observed at both low and high V/III ratios (see Figure S1a,b), however, one can note that the bright-field TEM image of the two staged sample presented in Figure S1c transitions to lower V/III flux ratios and favors more the frequent formation of twinning boundaries.

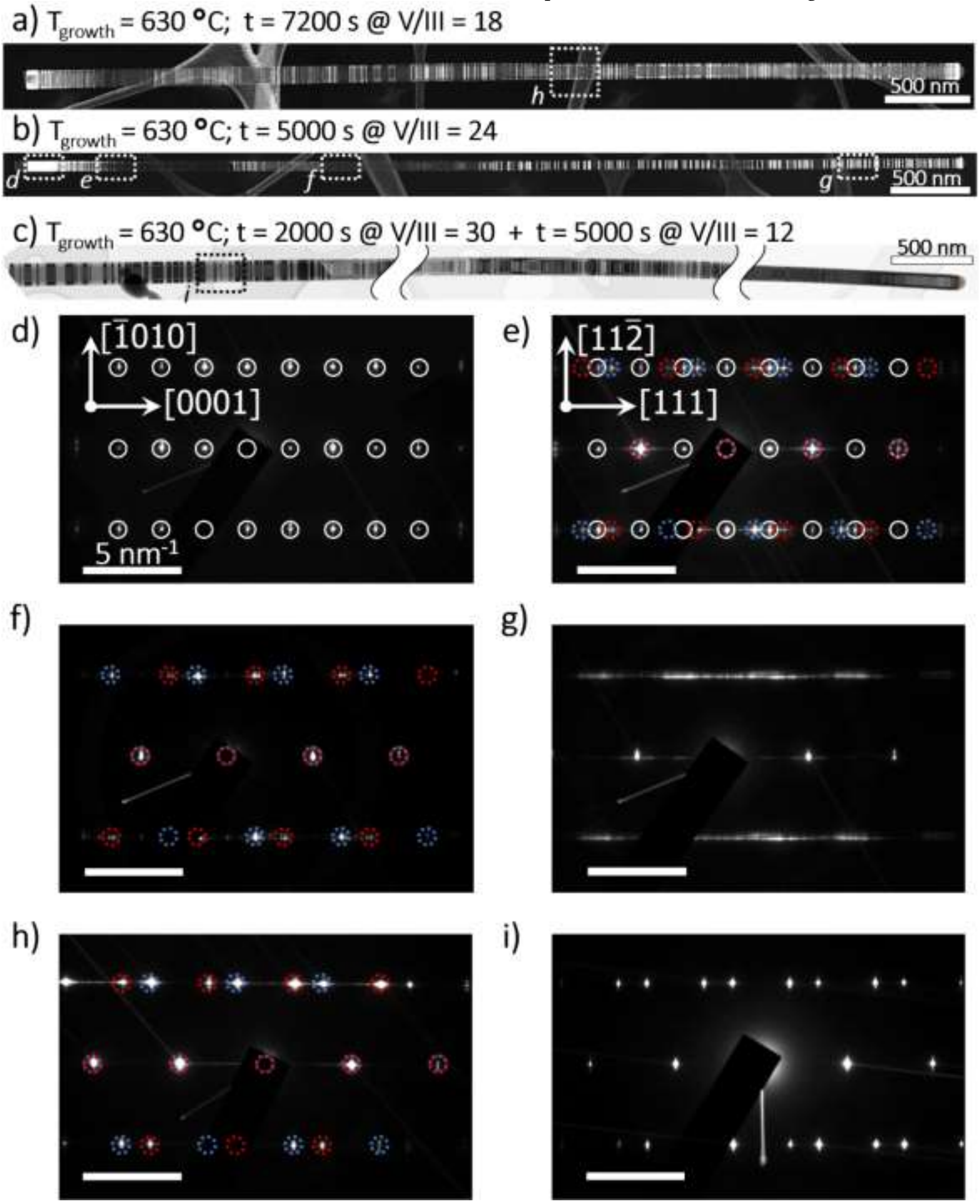

Figure S1. TEM images of the GaP NWs grown at the different growth conditions and transferred to a to carbon-coated copper TEM grid (**a**), (**b**) Dark-field TEM images with a WZ-diffraction contrast and (**c**) bright-field TEM images of the GaP NW grown via the proposed two-staged approach; (**d**–**i**) selective area electron diffraction patterns obtained from the NW areas with twinned ZB (f-i), WZ (d) and mixed crystal phase (e) structure schematically depicted with dashed lines in (**a**–**c**).

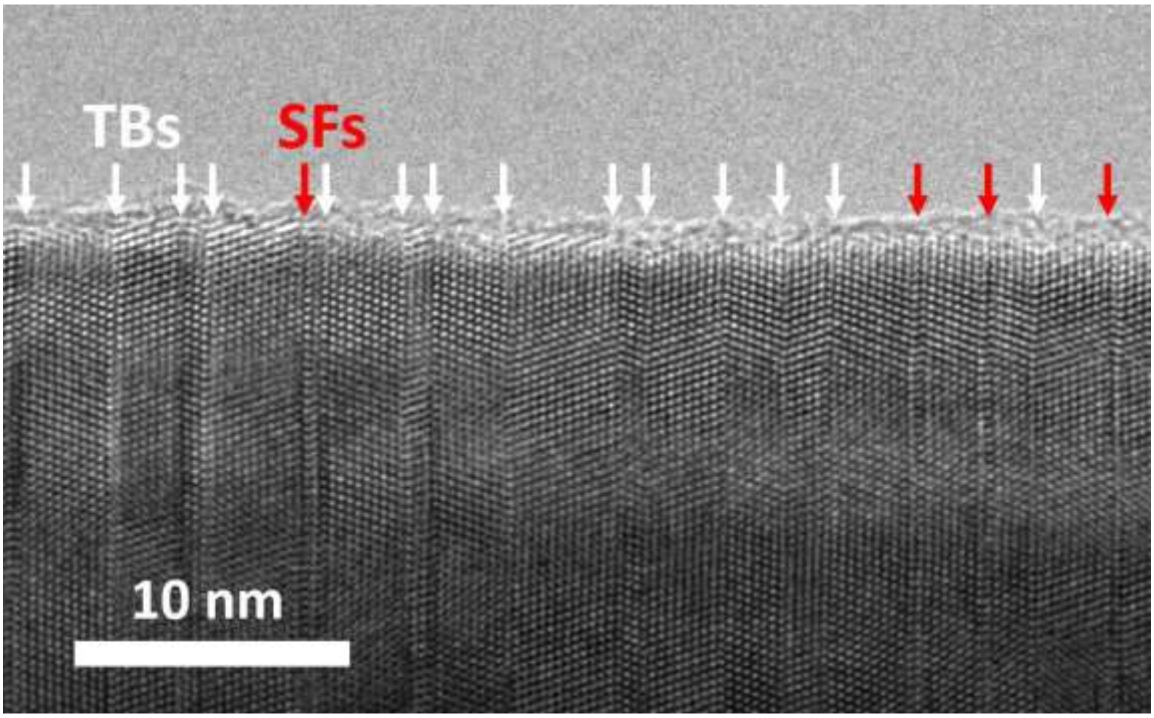

Figure S2. HRTEM images of the GaP NW demonstrating the atomic structure of planar defects. Twinning boundaries (TBs) and stacking faults (SFs) are marked by the white and red arrows, correspondingly.
